# Cancer Associated Fibroblast-Derived Hepatocyte Growth Factor Inhibits the Paclitaxel-Induced Apoptosis of Lung Cancer A549 Cells by Up-Regulating the PI3K/Akt and GRP78 Signaling on a Microfluidic Platform

**DOI:** 10.1371/journal.pone.0129593

**Published:** 2015-06-26

**Authors:** Li Ying, Ziwei Zhu, Zhiyun Xu, Tianrui He, Encheng Li, Zhe Guo, Fen Liu, Chunmeng Jiang, Qi Wang

**Affiliations:** 1 Department of Gastroenterology, the Second Hospital of Dalian Medical University, Dailan, China; 2 Department of Respiratory, the Second Hospital of Dalian Medical University, Dailan, China; 3 School of Medicine, Shanghai Jiao Tong University, Shanghai, China; University of Quebec at Trois-Rivieres, CANADA

## Abstract

Tumor stroma and growth factors provide a survival environment to tumor cells and can modulate their chemoresistance by dysregulating several signal pathways. In this study, we fabricated a three-dimensional (3D) microfluidic chip using polydimethylsiloxane (PDMS) to investigate the impact of hepatocyte growth factor (HGF) from cancer-associated fibroblasts (CAF) on the Met/PI3K/AKT activation, glucose regulatory protein (GRP78) expression and the paclitaxel-induced A549 cell apoptosis. With a concentration gradient generator, the assembled chip was able to reconstruct a tumor microenvironment *in vitro*. We found high levels of HGF in the supernatants of CAF and the CAF matrix from the supernatants of activated HFL1 fibroblasts or HGF enhanced the levels of Met, PI3K and AKT phosphorylation and GRP78 expression in A549 cells cultured in a 3D cell chamber, which was abrogated by anti-HGF. Inhibition of Met attenuated the CAF matrix-enhanced PI3K/AKT phosphorylation and GRP78 expression while inhibition of PI3K reduced GRP78 expression, but not Met phosphorylation in A549 cells. Inhibition of GRP78 failed to modulate the CAF matrix-enhanced Met/PI3K/AKT phosphorylation in A549 cells. Furthermore, inhibition of PI3K or GRP78 enhanced spontaneous and paclitaxel-induced A549 cell apoptosis. Moreover, treatment with the CAF matrix inhibited spontaneous and medium or high dose of paclitaxel-induced A549 cell apoptosis. Inhibition of PI3K or GRP78 attenuated the CAF matrix-mediated inhibition on paclitaxel-induced A549 cell apoptosis. Our data indicated that HGF in the CAF matrix activated the Met/PI3K/AKT and up-regulated GRP78 expression, promoting chemoresistance to paclitaxel-mediated apoptosis in A549 cells. Our findings suggest that the microfluidic system may represent an ideal platform for signaling research and drug screening.

## Introduction

The development of chemoresistance is a major obstacle for the successful treatment of non-small cell lung cancer (NSCLC)[1]. Many factors may contribute to the development of drug resistance in cancer and the genetic or epigenetic mutation of cancer cells is the most common and best-studied mechanism underlying drug resistance [2]. Recent studies have shown that tumor microenvironment provides a protective niche for cancer cells from drug treatment and eventually leads to chemoresistance [3–5]. Hence, identification of growth factor-mediated pro-survival pathways and targeting the members of the pathways have shifted the therapeutic paradigm from a chemotherapeutic to a target-specific approach in NSCLC [6, 7]. Cancer-associated fibroblasts (CAF) in the tumor stroma are crucial for the chemoresistance of cancer cells. CAF can produce growth factors that provide pro-survival signaling to the adjacent tumor cells in a paracrine manner [8, 9]. Hepatocyte growth factor (HGF) is one of the growth factors produced by CAF in tumor microenvironment and can bind its receptor of Met and activate the downstream phosphoinositide 3-kinase (PI3K)/AKT, which regulate chemoresistance and cell apoptosis [8]. This suggests that the HGF/Met and downstream PI3K/AKT signaling may be potential therapeutic targets against chemoresistant cancers. While dozens of inhibitors targeting the CAF-mediated pathways are currently under clinical studies, the efficacy of these inhibitors is clinically disappointing due to their poor specificity and severe adverse effects and functional overlap of many kinases [10].

Tumor microenvironment can trigger the stress response during cancer cell growth, leading to the accumulation of unfolded and/or misfolded proteins in the endoplasmic reticulum (ER) lumen, which subsequently provokes the unfolded protein response (UPR) [11]. Glucose-regulated protein 78 (GRP78) is an UPR protein, and is a master regulator of the UPR. Traditionally, GRP78 is recognized as a protective chaperone protein that supports cell survival by limiting the accumulation of unfolded proteins, facilitating calcium binding, and regulating transmembrane ER inducers [12]. Recent studies have shown that GRP78 is up-regulated in several tumors refractory to chemotherapy, like breast cancers, prostate cancers and hematologic cancers [13–15]. Our previous study has found increased levels of GRP78 expression by human small cell lung cancer NCI-H446 cells and that GRP78 inhibition re-sensitizes the cells to VP-16 [16]. These observations indicate that the up-regulated GRP78 expression in tumors is associated with the development of chemoresistance. However, the mechanisms underlying the up-regulated GRP78 expression in tumor cells remain largely unknown. Lately, it was reported that growth factor signaling could be coupled to ER chaperone expression. For example, the insulin-like growth factor-1 (IGF-1) signaling augmented the GRP78 expression in mouse livers via the PI3K/AKT/mTOR pathway, but not the canonical UPR [17]. Given that HGF can activate the PI3K/AKT signaling, we hypothesize that HGF may regulate the GRP78 expression by activating the PI3K/AKT signaling and GRP78 may be the key factor for development of chemoresistance in tumor cells.

It is well known that tumor microenvironment is crucial for cancer to respond to chemotherapy. Currently, many studies employ a three-dimensional (3D) synthetic culture system to mimic endogenous microenvironment [18]. Unfortunately, conventional 3D cultures commonly perform in multi-well plates (i.e., Transwell assay), and are difficult for high-throughput screening [19]. In addition, these culture platforms are all end-point assays, and are not amenable to real-time observation. The microfluidic-based cell culture system has many advantages in real time high-throughput screening due to distinct properties of cell-culture practices [18, 20, 21]. Typically, microfluidic technology enables the actuation of fluids and manipulation of bioparticles (e.g. cancer cells) at a microscale, which is cost-effective and minimal analysis time, and has a high sensitivity and resolution. The continual flow of laminar fluid in ultralow dimensions provides more efficient and accurate mass delivery to cancer cells [21]. Moreover, continuous nutrient supplies and waste removal through microchannel perfusion allow the cells to behave more closely to *in vivo* and to maintain the functionality longer. Most importantly, cancer cells in the microfluidic-based 3D scaffolding structure have diverse properties, similar to that of intact tumors [22–24]. Accordingly, we generated an integrated microfluidic culture chip, which exploited a double-layer 3D perfusion cell culture format to better mimic the complex nature of the tumor microenvironment. This system should allow to observe the real-time interaction of cancer cells with stromal cells and the dynamic changes in cellular signaling as well as drug responses.

In this study, we examined the impact of CAF or HGF on the Met/PI3K/AKT phosphorylation, GRP78 expression and paclitaxel-induced apoptosis in human non-small cell lung cancer A549 cells cultured in the 3D matrix. We found that neither culture mode nor matrix contents in the microfluidic platform promoted the proliferation of A549 cells. The CAF or HGF induced the Met/PI3K/AKT phosphorylation and up-regulated GRP78 expression in A549 cells, which were abrogated by treatment with anti-HGF. Furthermore, CAF inhibited the paclitaxel-induced A549 cell apoptosis while inhibition of PI3K or GRP78 enhanced spontaneous and paclitaxel-induced A549 cell apoptosis. Our data indicated that HGF in the CAF activated the Met/PI3K/AKT and up-regulated GRP78 expression, contributing to chemoresistance to paclitaxel in A549 cells in vitro and in vivo.

## Materials and Methods

### Microfluidic chip fabrication

**Fig 1:**
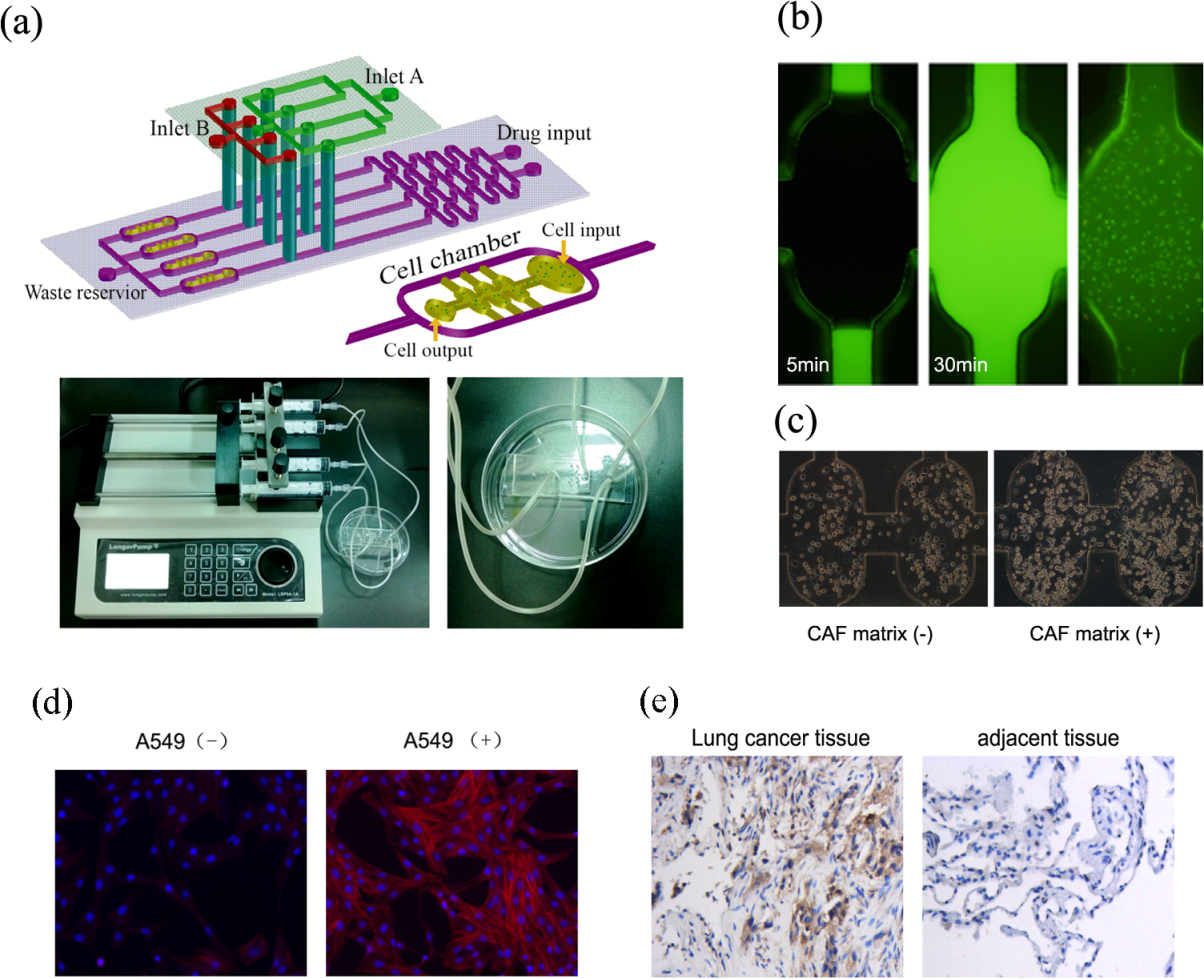
The design and validation of a 3D culture microfluidic chip. (a)The schematic design of the microfluidic chip with CGG and downstream cell chambers (the upper panel) and the fabricated chip with pumping machine (the lower panel). (b)The diffused Rh-123 in the 3D chamber within 30 min and >95% cells were viable (green). Magnification ×100. (c) The morphological features of A549 cells in the 3D chamber without or with CAF matrix. The white arrows indicate apoptotic cells. (d)The α-SMA immunofluorescence assay of HFL1 cells. HFL1 cells induced by A549 medium showed a positive α-SMA staining (right) compared to the untreated HFL1 (left). Magnification ×400. (e) Immunohistochemistry assay for lung cancer tissues. The expression of α-SMA protein in the lung cancer tissues is higher than that in adjacent tissues. Magnification ×200.

The schematic design of microfluidic device with a two-layer structure is shown in Fig 1A. The lower layer consisted of a combination of a linear concentration gradient generator (CGG) and four downstream parallel cell culture units with two oval-shape modules. The CGG had two inlets (a diameter of 1.5 mm) for medium and drug solution perfusion and corresponding cascade microchannels (10 mm × 200 μm × 100 μm). The CGG utilized diffusive mixing to generate a mixture of the two inlets at the mixing microchannels. The concentration interval from the channel 1 to channel 4 generated by CGG in theory is (drug concentration_max_—drug concentration_mix_)/3, which had been demonstrated in our previous study [25]. The dimensions of each chamber used for cell culture were 800 μm (length) × 400 μm (width) × 100 μm (height). The inlet and outlet diameters of cell chamber were 0.6 mm. Accordingly, the mixture of cell-basement membrane extracts (BME) was seeded in the cell culture chamber, where cells were cultured in 3D. The excess mixture was effused from a cell outlet. The upper PDMS layer possessed two inlets (a diameter of 1.5 mm) and multiplexed perfusion channels (200 μm in width and 100 μm in height). Hence, soluble factors, fibroblast-secreted growth factors and drugs flowed to the cell chambers on the lower layer. The two layers were combined through the exactly matched holes inside the channels of upper and lower layers by using a stereomicroscope with the reference marks.

The chip was fabricated with polydimethylsiloxane (PDMS, Sylgard 184, Dow Corning, Midland, MI, USA) by standard soft lithography method [26]. Briefly, silicon templates were prepared by spin-coating a layer of SU8-2035 negative photoresist (Microchem, Newton, MA, USA) onto a glass wafer and patterned by photolithography. The PDMS base and curing agent were mixed thoroughly (10:1 in mass), degassed under vacuum, and poured onto the master. The polymer was oven-cured for 1 h at 80°C. After cooling, the PDMS layer was gently peeled from the master and trimmed to size. Holes were punched out of the PDMS to form reservoirs for liquid introduction. The resulting PDMS structures were oxidized in oxygen plasma (150 mTorr, 50 W, 60 s) for irreversible chemical bonding to glass slides before connecting to fluidic components. Cells and culture media were introduced through MS26 injection pumps, pushing the plunger of a syringe forward at an accurately controlled rate. The fluid flow rate was controlled at 10 mm/24 hours. Prior to co-culture assay, the microfluidic device was dipped in double-distilled water and UV-sterilized for 30 min. The culture chambers were filled with poly-l-lysine solution (0.01%, m/v) (Sigma–Aldrich, St. Louis, MO, USA) for 1 h to coat their inner surface.

### Cell culture and treatment

Human lung cancer A549 and fibroblast HFL1 cells were purchased from the Cell Bank of Type Culture Collection of Chinese Academy of Sciences (Shanghai, China). A549 and HFL1 cells were maintained in PRMI 1640 and IMDM (Cellgro, USA) supplemented with 10% fetal bovine serum (FBS, Hyclone, Logan, USA) and antibiotics (penicillin, 100 U mL^-1^; streptomycin 100 μg mL^-1^) at 37°C in a humidified atmosphere of 5% CO_2_, respectively. The cells were grown to approximately 80% confluence and collected for experiments. Furthermore, A549 cells (1000 cells/chamber) were cultured in maintenance medium alone, a mixture of maintenance medium and CAF in the presence or absence of human HGF neutralizing antibody (20 μg/ml; R&D systems, USA), or maintenance medium containing HGF (40 ng/ml, Sigma, USA) for 48h. Alternatively, the cells were cultured in a mixture of maintenance medium and CAF for 24 h and then cultured in the same medium or the medium containing an inhibitor for Met (SU11274, 5 μmol/L; Sigma, USA), PI3K/AKT1 (LY294002, 20 μmol/L; Beyotime, China) or GRP78 (BAPTA-AM-A1076, 20 μmol/L; Sigma, USA) for another 24 h.

### CAF culture medium preparation

The CAF culture medium was prepared, according to a modified method [27]. Briefly, HFL1 cells were cultured in a mixture (1:1) of maintenance medium and the supernatants of cultured A549 cells in semiconfluent conditions for a minimum of 20 passages prior to matrix production. The supernatants from cultured HFL1 were harvested at different time point and centrifuged at 2000 × g, stored at -70°C. The concentrations of HGF in the harvested supernatants were determined and the supernatants were used as CAF culture medium.

### Determination of HGF concentration in CAF culture medium

The levels of HGF in the supernatants were determined by ELISA using a specific kit, according to the manufacturers’ instruction (Immunis HGF EIA, Japan). The samples were tested in triplicate and the concentrations of HGF were determined, based on the standard curve established using recombinant HGF provided.

### Microfluidic culture and viability analysis

A549 cells were cultured in 3D microfluidic chips, as our previous work [28]. Briefly, A549 cells in exponential growth phase were trypsinized and re-suspended in maintenance medium. The cells at 10^6^ cells/mL were mixed with BME at 1:1 and injected into the 3D cell culture chambers through cell inlet, followed by cultured at 37°C in 5% CO_2_ and 95% relative humidity. Fresh media and HGF-contained CAF were introduced from inlet A on the upper layer and transmitted to A549 culture chamber on the lower layer. The cells in the cell chambers were stained with Rh-123 (2 μg/mL, Sigma, USA) at 37°C for 30 min, washed with PBS and examined under an inverted fluorescent microscope to determine their growth pattern.

### Immunofluorescence assay

The specific biomarker of CAF, α-SMA and the Met/PI3K/AKT activation and GRP78 expression induced by the CAF in A549 cells were determined by immunofluorescence assay. The cultured A549 cells in the chambers were washed with PBS, were fixed with 4% paraformaldehyde for 15 min and then permeabilized in 0.1% Triton X-100 for 25 min. After being blocked with goat sera at 37°C for 30 min, the cells in the chambers were treated with α-SMA (1:1000, Abcam, UK) anti-p-Met (Try1234/1235; 3D7, 1:200), anti-p-PI3K p85 (Y458, 1:100), anti-p-Akt1 (Thr 308, 1:100) and anti-GRP78 (1:100, Cell Signaling, USA) at 4°C overnight. After being washed, the bound antibodies were detected with suitable Dylight 594 red-conjugated or FITC green-conjugated secondary antibodies and photo imaged under a fluorescent microscope.

### Western blot assay

The relative levels of Met, PI3Kp85, AKT1 phosphorylation and GRP78 expression in A549 cells were determined by Western blot. The cells were treated, as described above, harvested, and lyzed in ice-cold RIPA buffer with fresh 10 mg/ml phenylmethanesulphonyl fluoride and 1% (V/V) cocktail protease inhibitor (Sigma, USA) for 30 min. The cell lysates were briefly sonicated and centrifuged, and the concentrations of proteins in cell lysates were determined by BCA protein assay kit (Pierce, Thermo Fisher Scientific, USA). The cell lysates (100 μg/lane) were separated on 12.5% gels by sodium dodecyl sulfate polyacrylamide gel electrophoresis (SDS-PAGE) and transferred onto nitrocellulose membranes (Millipore, USA). After being blocked with 5% fat-free dry milk, the membranes were incubated with antibodies against Met, p-Met, GRP78 (Sigma), PI3Kp85, p-p85 (Beyotime, China), AKT1, p-AKT1 (Cell Signal Technology) and β-actin (Santa Cruz Biotechnology) [29]. The bound antibodies were detected with horseradish peroxidase (HRP)-conjugated second antibodies and visualized using enhanced chemiluminescence reagents (Amersham Biosciences, USA). The relative levels of each protein to the internal control were quantified using the Gel-pro4.0 software (Media Cybernetics).

### Immunohistochemistry

The expression of α-SMA in the lung cancer tissues and adjacent tissues was assessed by immunostaining of α-SMA protein, with an antibody against α-SMA and the SP-9000 HistostainTM-Plus Kit (ZYMED, MA, USA). Specifically, staining of α-SMA expression was visualized as a brown color in the cytoplasm and estimated by averaging the number of positive-stained cells under 10 high-power vision fields.

### Analysis of A549 cell apoptosis

The impact of modulating the HGF/Met/PI3K/AKT signaling and GRP78 expression on paclitaxel-induced A549 cell apoptosis was determined by a fluorescent assay. Briefly, A549 cells (1000 cells/chamber) were cultured in the chambers for 24h to reach 50% confluence and the cells were exposed to different concentrations (0–4 μM) of paclitaxel in maintenance medium or the mixture of maintenance medium and CAF in the presence or absence of an inhibitor for PI3K or GRP78 (as the concentrations described above) for another 24 h. The drug and inhibitors were introduced into the microfluidic device using a syringe pump through the two upstream drug inlets of the CGG on the lower layer and through the inlet B on the upper layer, respectively. Subsequently, the cells were washed and, the percentages of apoptotic cells were determined using Hochest33342 and PI staining. At least 500 cells from each group of 10 fields selected randomly were analyzed.

### Nude mice in vivo xenograft model

BALB/c-nu nude mice (6 weeks old) were purchased from the Laboratory Animal Centre, Dalian Medical University, and the animal study was approved by the Life Sciences Institutional Review Board of Dalian Medical University. The A549 cells (5 × 10^7^/ml) in 200 μL of PBS were injected into the back of nude mice. The xenograft nude mice were divided into different groups: control group, HGF group, HGF+PI3K inhibitor group, HGF+GRP78 inhibitor group. HGF (1μg, Preprotech) in 200 μL of PBS was injected around the tumor on 15 d following tumor inoculation. PI3K inhibitor LY294002 was administered at 10mg/kg/d by intraperitoneal injection and GRP78 inhibitor BAPTA-AM-A1076 was administered at 5mg/kg/d by intraperitoneal injection respectively. Treatment started 30 d following tumor inoculation. Then all nude mice in different groups were injected with paclitaxel (25mg/kg) around the tumors for three times on 45 d following tumor inoculation. Tumor size was measured every five days. After 60 days, the tumor size was calculated according to the formula V = L × W^2^ / 2.

### Statistical analysis

All experiments were performed for at least three times. Data are expressed as the means ± S.D. The difference among groups was analyzed by ANOVA and post hoc Fisher's least significant difference using the SPSS17.0 for Windows software. A *P* value of <0.05 was considered statistically significant.

## Results

### Performance of the integrated microfluidic device

Using established polymer microfabrication technology, we created a microfluidic 3D culture device to test the drug sensitivity of A549 cells by reconstructing a tumor microenvironment *in vitro* with continuous nutrient supplies and CAF-derived matrix. Experimentally, the CGG was able to generate the correct mixture of the solutions that had been injected through the two drug inlets. As seen in Fig 1A, the CGG consisted of a two-level mixing stage and each level could mix the two input fluids within the first couple of turns. The CGG was connected to four 3D cell culture chambers by oval microchannels. Following injection with Rh-123 solution, Rh-123 diffused through the entire BME of 3D cell culture chamber within 30 min (Fig 1B). Subsequently, Rh-123 was sequestered by the active mitochondria of more than 95% living cells in the 3D culture chambers. With this design, the cells in the 3D culture chambers could be treated with drugs at various concentrations generated by the CGG, and with a specific protein or growth factor.

To assess the role of CAF in regulating the proliferation of tumor cells, A549 cells were cultured in maintenance medium with, or without, CAF matrix for 3 days. We found that culture of A549 cells in the CAF matrix obviously increased the numbers of viable cells, suggesting that the CAF matrix promoted the proliferation of A549 cells in our experimental system. Morphologically, A549 cells became notably rounded or amoeboid-like in both culture conditions. However, A549 cells spontaneously formed multicellular clusters in culture with CAF matrix compared to scattered distribution in the cells cultured without CAF matrix (Fig 1C).

### Transformation from fibroblast to CAF

In order to obtain the CAF for the in vitro study, we induced CAF from fibroblast. In brief, human lung cancer cell line A549 was loaded in the upstream of cell chambers in the chip and then human lung fibroblast cell line HFL1 was loaded in the downstream. The A549 medium flows in the HFL1 cell chambers and the cell culture media were continuously supplied for 24h under a pump. The α-SMA immunofluorescence assay indicated that HFL1 induced by A549 medium showed a positive α-SMA staining compared to the untreated HFL1 (Fig 1D). Also we confirmed the phenotype of CAF in vivo, the α-SMA immunohistochemistry showed that α-SMA protein was higher expressed in lung cancer tissue than that in adjacent lung cancer tissue (Fig 1E).

### HGF in the CAF induces the Met/PI3K/AKT activation in A549 cells

**Fig 2:**
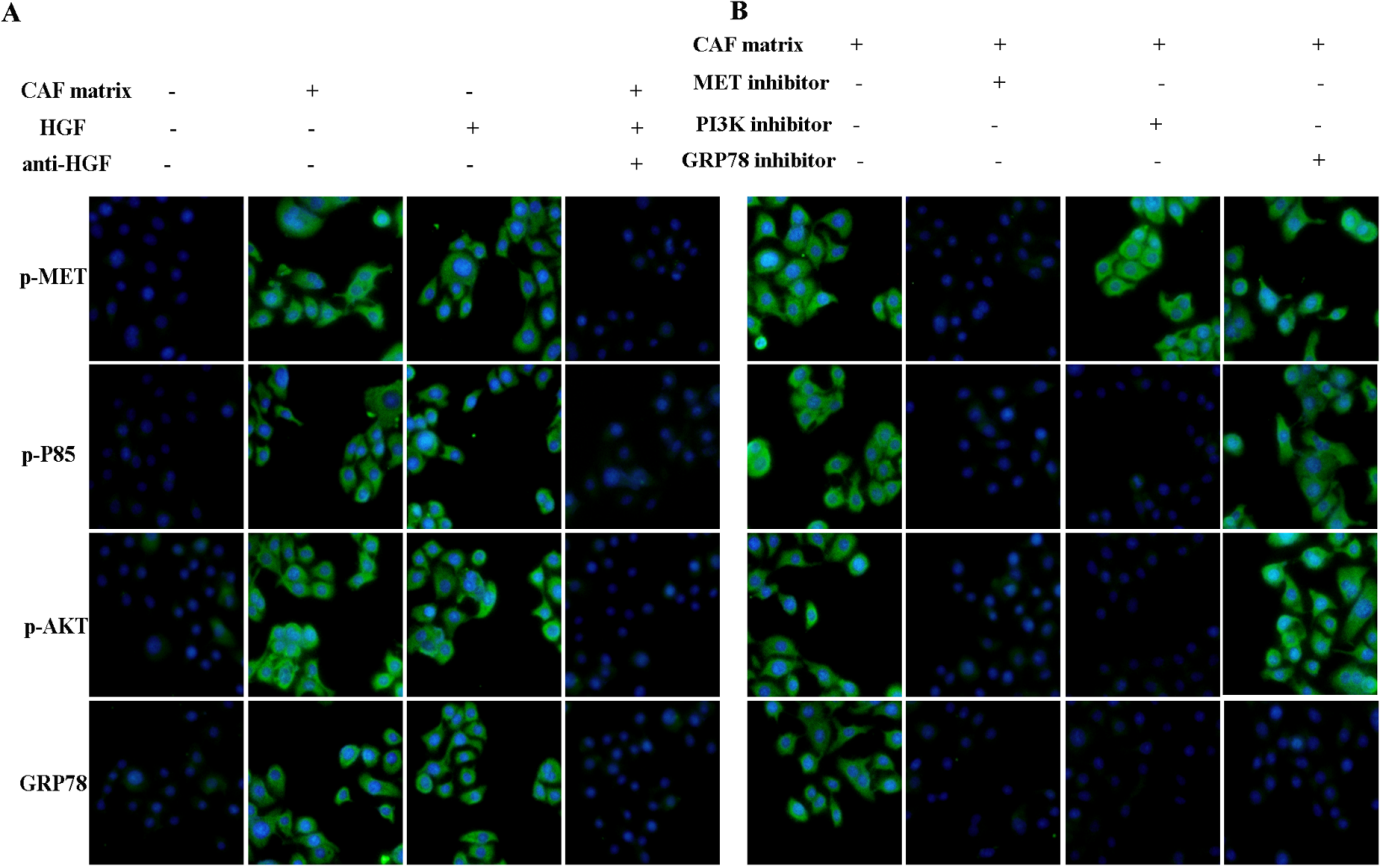
Immunofluorescent analysis of the MET/PI3K/AKT activation and GRP78 expression on the microfluidic chip. A549 cells were cultured in triplicate in the maintenance medium alone, mixed with the CAF matrix in the presence or absence of anti-HGF or containing 40 ng/ml of human HGF in the 3D chambers for 48h. The cells were stained with the indicated FITC-conjugated antibodies, and examined under a fluorescent microscope. Furthermore, the cells were cultured in the mixture of maintenance medium and CAF matrix in the presence or absence of an inhibitor for c-Met, PI3K or GRP78 for 48h and stained as described above. Data are representative images (magnification x 200) from three separate experiments. (A)The CAF matrix or HGF enhances the c-Met/PI3K/AKT activation and GRP78 expression in A549 cells. (B)The effect of an inhibitor of c-Met, PI3K or GRP78 on the CAF-enhanced c-Met/PI3K/AKT activation and GRP78 expression in A549 cells.

To investigate the potential mechanisms underlying the effect of tumor stroma on cancer cells, we examined the Met, PI3K and AKT phosphorylation in A549 cells cultured in the 3D chambers by immunofluorescence assay. As expected, A549 cells cultured in CAF matrix displayed higher levels of anti-phosphorylated Met, PI3K and AKT and anti-GRP78 staining compared to those cultured without CAF matrix (Fig 2A). Previous studies have shown that tumor stromal cells, such as fibroblasts, can secrete HGF that can promote the proliferation of cancer cells [28]. We questioned whether HGF in the CAF promoted the Met, PI3K and AKT activation and up-regulated GRP78 expression in A549 cells. To address this question, we first determined the concentrations of HGF in the CAF collected from activated HLF1 cells that had been cultured for varying periods. We found that the levels of HGF in the supernatants of cultured HLF1 cells gradually increased with time and peaked (400 ng/2×10^5^ cells) at 72 h post culture. However, there was no detectable level of HGF in the supernatants of cultured A549 cells (data not shown). Next, we tested whether HGF mediated the Met/PI3K/Akt activation and up-regulated GRP78 expression in A549 cells. We found that addition of 40 ng HGF to maintenance medium also increased anti-phosphorylated Met, PI3K and AKT as well as anti-GRP78 staining in A549 cells. More importantly, addition of human HGF neutralizing antibody (20 μg/ml) into the mixture of maintenance medium and CAF containing almost prevented anti-phosphorylated Met, PI3K and AKT as well as anti-GRP78 staining in A549 cells.

**Fig 3:**
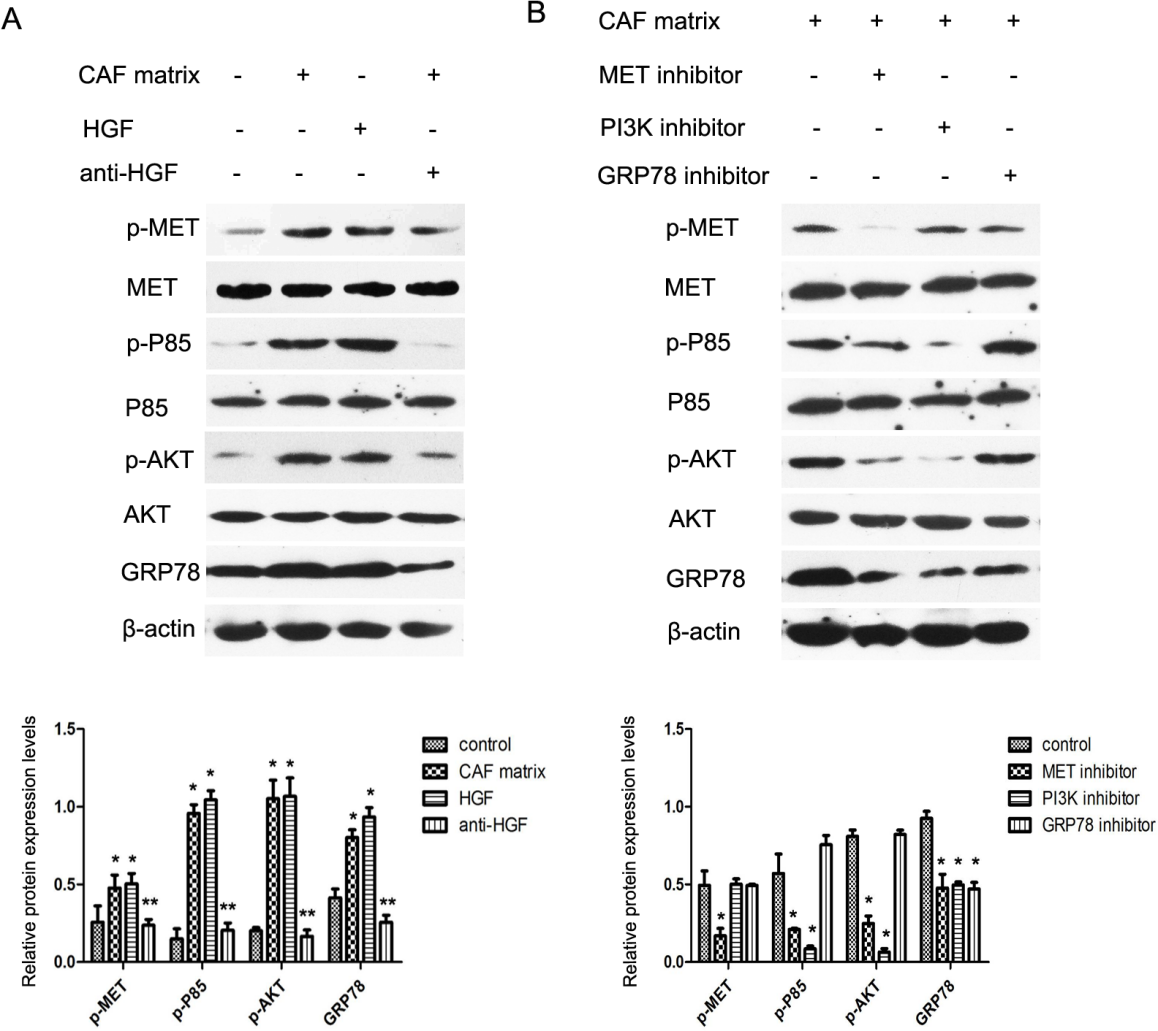
Western blot analysis of the c-Met/PI3K/AKT activation and GRP78 expression in A549 cells. A549 cells were cultured in the condition as described above and the relative levels of phosphorylated Met, PI3Kp85, AKT and GRP78 expression in the different groups of cells were characterized by Western blot assays and quantified. Data are representative images and expressed as the means ± SD of each protein in individual groups of cells from three separate experiments. *P<0.05; **P<0.01 vs. the controls.

In addition, treatment with an inhibitor for Met attenuated CAF-mediated Met phosphorylation and reduced the levels of PI3Kp85 and AKT phosphorylation and GRP78 expression in A549 cells (Fig 2B). Furthermore, treatment with a PI3K inhibitor reduced the CAF-mediated PI3Kp85 and AKT phosphorylation and GRP78 expression, but did not change the CAF-mediated Met phosphorylation in A549 cells. Moreover, treatment with a GRP78 inhibitor only down-regulated CAF-mediated GRP78 expression, but did not modulate the CAF-mediated Met, PI3Kp85 and AKT phosphorylation in A549 cells. A similar pattern of the CAF-mediated Met/PI3K/AKT phosphorylation and GRP78 expression in A549 cells was observed by Western blot assay (Fig 3). Collectively, these data indicated that HGF in the CAF stimulated the Met/PI3K/AKT activation and up-regulated GRP78 expression in A549 cells.

### HGF in the CAF matrix induces hyposensitivity to paclitaxel in A549 cells

**Fig 4:**
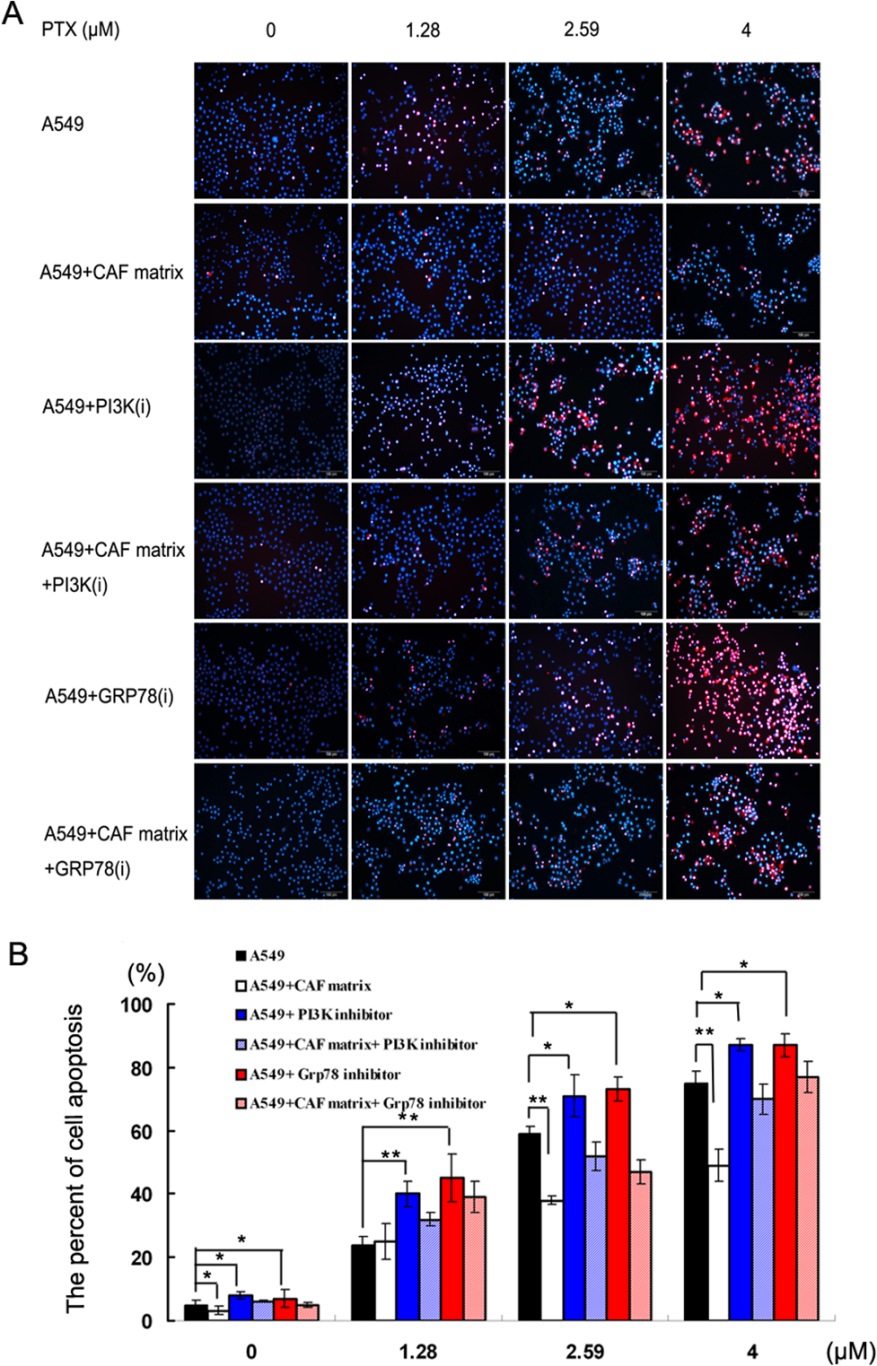
The percentages of apoptotic A549 cells. A549 cells were cultured in maintenance medium for 24h and continually cultured in triplicate in the same medium or mixture of maintenance medium and CAF matrix in the presence or absence of the PI3K or GRP78 inhibitor on the 3D chambers for 24h. Subsequently, the cells were stained with Hochest33342 and PI, imaged and the percentages of apoptotic A549 cells were counted. Data are representative images (Fig 4A) (magnification x 200) or expressed as the means ± SD of the percentages of apoptotic cells in individual groups of cells from three separate experiments (Fig 4B). *P<0.05; **P<0.01.

We next examined the impact of HGF in the CAF on paclitaxel-induced A549 cell apoptosis in 3D chambers. The culture media and paclitaxel at 4 μM were simultaneously introduced through the two upstream inlets of the CGG. Consistent with theoretical calculations, the concentrations of paclitaxel in the experimental channels 1–4 were 0.00, 1.28, 2.59, 4.00 μM, respectively. A549 cells were cultured in maintenance medium or the mixture of maintenance medium and CAF for 24h and the cells were exposed to different concentrations of paclitaxel in the presence or absence of the inhibitor for PI3K or GRP78 for another 24h. The apoptotic cells were determined by staining with Hochest33342 and PI (Fig 4A). Quantitative analyses revealed that low frequency of spontaneous apoptotic A549 cells was detected when the cells were cultured in maintenance medium (Fig 4B). Culture of A549 cells in the mixture of maintenance medium and CAF matrix significantly reduced the percentages of apoptotic cells (p<0.05). These data suggested that HGF in the CAF inhibited spontaneous A549 cell apoptosis in vitro. Furthermore, treatment with different concentrations of paclitaxel induced A549 cell apoptosis in a dose-dependent manner. In addition, culture of A549 cells in the mixture of maintenance medium and CAF matrix significantly reduced the percentages of A549 cell apoptosis induced by a medium or high dose of paclitaxel (p<0.01). Therefore, HGF in the CAF inhibited spontaneous and paclitaxel-induced A549 cell apoptosis in vitro.

### The PI3K/AKT or GRP78 inhibitor enhances the sensitivity of A549 cells to paclitaxel

Given that the pro-survival role of the PI3K/AKT activation is associated with chemoresistance of cancers [30, 31], we examined whether the blockade of PI3K/AKT could reverse the HGF-induced resistance to paclitaxel. Clearly, treatment with the inhibitor for PI3K or GRP78 significantly increased the frequency of spontaneous and paclitaxel-induced A549 cell apoptosis in vitro (p<0.05 or p = 0.02, Fig 4B). Moreover, culture of A549 cells in the mixture of maintenance medium and CAF matrix containing the inhibitor for PI3K increased the percentages of apoptotic cells, as compared with the cells cultured in the mixture of CAF containing different concentrations of drug in the absence of the inhibitor in vitro (38±3.0% vs. 20±1.4%in the 1.28 μM paclitaxel subgroup, 69±3.9% vs. 44±5.5% in the 2.59 μM subgroup, and 79±5.1% vs. 62±2.5% in the 4 μM subgroup, *p*<0.05, Fig 4B). These data indicated that the blockade of PI3K/AKT augmented chemotherapeutic sensitivity of A549 cells. Similarly, treatment with the GRP78 inhibitor also increased the percentages of paclitaxel-induced apoptotic A549 cells cultured in the mixture of CAF, as compared with that in the cells cultured in the same medium without the inhibitor (40±2.5% vs. 20±1.4% in the 1.28 μM paclitaxel subgroup; 62±2.7% vs. 44±5.5% in the 2.59 μM subgroup; and 80±1.9% vs. 62±2.5% in the 4 μM subgroup, p<0.05 for all). Finally, treatment with both the PI3K and GRP78 inhibitors further elevated the percentages of paclitaxel-induced apoptosis in A549 cells cultured in maintenance medium (90%, 95% and 100% in the 1.28, 2.59 and 4 μM paclitaxel subgroups, respectively). Together, these data indicated simultaneous inhibition of the PI3K and GRP78 enhanced the sensitivity of A549 cells to paclitaxel.

### The validation in in vivo xenograft model

**Fig 5:**
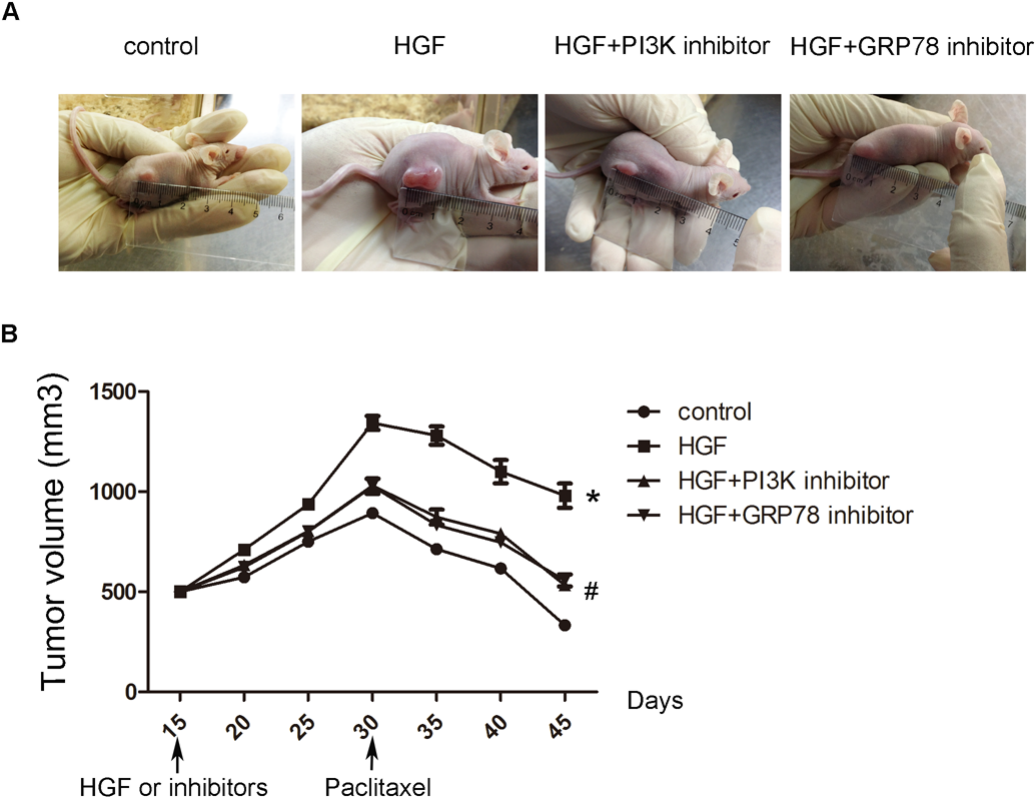
The validation in in vivo xenograft model. (A) Representative images of tumor size in different groups of nude mice on the 60th day after treatment. (B) Growth curves in different groups of nude mice. *P<0.05 vs control group, ^#^ *P*<0.05 vs HGF group.

In order to confirm the results from 3D in vitro model, we used in vivo xenograft model to validate. As shown in Fig 5A, the tumor size in HGF group was significantly bigger than that in control group. While PI3K and GRP78 were blocked, the tumor size was reversed. Following treatment with therapeutic drug with paclitaxel, all the tumor volumes in different groups significantly reduced, as compared with that beginning with treatment. However, the mean percentage (27%) of tumor volume reduction in the HGF-treated tumors was obviously less than that of the control (62.7%), HGF+PI3K inhibitor (48.3%) and HGF+GRP78 inhibitor-treated tumors (45.8%) (Fig 5B). The results indicated that HGF induced A549 drug resistance, and PI3K and GRP78 were key factors in the above resistance. Compared to the *in vitro* results, the similar results were found in *in vivo* system.

## Discussion

Tumor microenvironment is composed of not only cancer cells but also various stromal cells, such as fibroblasts, vascular cells, and inflammatory cells [32]. Fibroblasts can secrete extracellular matrix (ECM) components, growth factors and chemokines, and regulate the cancer development and chemoresistance, besides their direct interaction with cancer cells. HGF can be produced by stromal fibroblasts in a tumor microenvironment and is responsible for activation of the HGF/c-Met signaling during the advancement of lung cancer [33]. Furthermore, HGF plays a definitive role in both intrinsic and acquired resistance to anti-tumor drug by increasing the PI3K/AKT signaling, an important survival pathway in NSCLC [8]. In this study, we examined HGF production and found high levels of HGF secreted by activated HFL1 cells, but not by A549 cells. Furthermore, culture with CAF matrix or HGF increased the levels of c-Met, downstream PI3Kp85 and AKT phosphorylation in A549 cells, consistent with previous studies [6, 34, 35]. More importantly, the up-regulated cMet/PI3K/AKT activation by the ACF matrix was almost completely abrogated by treatment with anti-HGF in A549 cells. Functionally, the CAF matrix attenuated spontaneous and a medium or high dose of paclitaxel-induced A549 cell apoptosis in vitro. Moreover, inhibition of PI3K not only enhanced spontaneous and paclitaxel-induced A549 cell apoptosis, but also reversed the CAF matrix-mediated inhibition by enhancing paclitaxel-induced A549 cell apoptosis in vitro. Our data support the notion that the CAF matrix, particularly for HGF, enhances the pro-survival PI3K/AKT activation and contributes to the chemoresistance of cancer cells.

A recent study has shown that IGF-1 can activate the PI3K/AKT pathway and up-regulate the ER chaperone GRP78 expression in mouse liver [17]. The increased GRP78 expression is associated with resistance to apoptotic triggers and chemotherapy in cancers [14]. We found that the CAF matrix or HGF enhanced GRP78 expression, accompanied by activating the Met/PI3K/AKT pathway in A549 cells (Figs 2 and 3). Furthermore, inhibition of Met, or PI3K attenuated the CAF matrix-up-regulated GRP78 expression in A549 cells. However, inhibition of GRP78 did not modulate the CAF matrix-enhanced Met/PI3K/AKT activation in A549 cells. These data suggest that GRP78 expression may be a downstream target of the PI3K/AKT pathway in A549 cells. Alternatively, HGF through its receptor of c-Met activates the PI3K/AKT pathway, which cross-talk with the UPR to up-regulate GRP78 expression. We are interested in further investigating whether the PI3K/AKT pathway can directly promote the GRP78 expression in cancer cells. More importantly, the up-regulated GRP78 expression resulted in chemoresistance to paclitaxel and inhibition of GRP78 enhanced paclitaxel-induced apoptosis in A549 cells, suggesting that GRP78 may be valuable for fibroblast-mediated inhibition of apoptosis. Conceivably, GRP78 may be a target for chemotherapy of lung cancer. Recently, combination of different molecules targeting different pathways has envisioned as an effective modality to treat drug-resistant tumors. We found that treatment with paclitaxel, the PI3K or GRP78 inhibitor alone induced A549 cell apoptosis and combination of them synergistically enhanced A549 cell apoptosis. Therefore, combination of paclitaxel with a GRP78 or PI3K inhibitor may be promising strategies for chemotherapy of lung cancer.

High-throughput analytical tools are crucial for screening many signal pathways simultaneously. Microfluidic platforms have been widely used for oncological researches, especially in cancer cell biology and antitumor drug discovery owing to their unique functionality of high-throughput and their remodeling of precise scaffolds [20, 36]. In this study, we employed a two-layer PDMS chip to culture cells with concentration gradient mixers and MS26 injection pumps to investigate complex, heterotypic cellular interactions, and importantly, to accomplish a rapid, efficient, and high-throughput drug sensitivity tests. Compared with the technical platforms of conventional cell culture platform, our microfluidic device presented its advantages in less sample and reagent consumption, shorter assay time, and higher sensitivity.

The multiplex microchannels allowed a normal fluid to flow, through which agents and growth factors secreted by fibroblasts could be transmitted to the A549 cell culture chamber. Considering an inherent limiting factor of shear stress under bulk fluid flow in microfluidic perfusion culture, which resulted in a detrimental effect on cells in the culture chambers, oval perfusion microchannels were molded to connect 3D cell culture chamber arrays. Because the effect of convection was greatly reduced in the microchannels, the transport of soluble molecules from the fluid delivery level into the cell culture level was governed almost exclusively by diffusion and the tumor cells inside were not only protected against direct convective flow, but also modulated by the upstream growth factors. This configuration mimics the physiological cell microenvironment that the central cell culture micro-well represents the “tissue space” with a fast convective “blood” flow through the outer channel, and diffusion dominated transport into the low flow “interstitial space”[37]. Furthermore, the performance of the integrated microfluidic gradient generator was validated to generate a gradient downward due to diffusive mixing. This characterization allowed the concentrations of the agents at each chamber to be known and such information would assist in designing the clinical experiments with suitable dosages. However, there are also some limitations in this study. For example, the sample loading should be improved in the microfluidic system. The uneven distribution of cells in the cell chambers should be optimized. Also the knock down or knock out cell lines should be used for the investigation of pathways.

In summary, our data indicated that the CAF matrix and the contained HGF enhanced the PI3K/AKT activation and GRP78 expression, and promoted chemoresistance to paclitaxel in A549 cells. Hence, HGF and these molecules may be targets for intervention of drug-resistant lung cancer and combination of targeting two or more molecules may be valuable for intervention of drug-resistant lung cancers.
